# A Systematic Meta-analysis of Immune Signatures in Patients With Acute Chikungunya Virus Infection

**DOI:** 10.1093/infdis/jiv049

**Published:** 2015-01-29

**Authors:** Terk-Shin Teng, Yiu-Wing Kam, Bernett Lee, Hapuarachchige Chanditha Hapuarachchi, Abeyewickreme Wimal, Lee-Ching Ng, Lisa F. P. Ng

**Affiliations:** 1 Singapore Immunology Network, Agency for Science, Technology and Research (A*STAR), Biopolis; 2 Environmental Health Institute, National Environment Agency, Singapore; 3 Department of Parasitology, Faculty of Medicine, University of Kelaniya, Ragama, Sri Lanka; 4 Institute of Infection and Global Health, University of Liverpool, United Kingdom

**Keywords:** chikungunya virus, acute infection, cytokines, chemokines, meta-analysis

## Abstract

***Background.*** Individuals infected with chikungunya virus (CHIKV) normally exhibit a variety of clinical manifestations during the acute phase of infection. However, studies in different patient cohorts have revealed that disease manifestations vary in frequency.

***Methods.*** Disease profiles between patients with acute CHIKV-infection and febrile patients without CHIKV were compared and examined to determine whether any clinical presentations were associated with the clinical outcome of CHIKV infection. Circulatory immune mediators profiles were then characterized and compared with data from 14 independent patient cohort studies. The particular immune mediator signature that defines acute CHIKV infection was determined.

***Results.*** Our findings revealed a specific pattern of clinical presentations of joint-specific arthralgia from this CHIKV cohort. More importantly, we identified an immune mediator signature dominated by proinflammatory cytokines, which include interferon α and γ and interleukin 2, 2R, 6, 7, 12, 15, 17, and 18, across different patient cohorts of CHIKV load associated with arthralgia.

***Conclusions.*** To our knowledge, this is the first study that associated levels of CHIKV load with arthralgia as an indicator of acute CHIKV infection. Importantly, our findings also revealed specific immune mediator signatures that can be used to better define CHIKV infection.

The reemergence of chikungunya virus (CHIKV) in the last decade, notably in the Indian Ocean islands, India, and Southeast Asia has inflicted millions to date [1–3]. Alarmingly, CHIKV transmission has been on the rise in the Western hemisphere in islands of the Caribbean, with close to 800 000 cases documented recently [4, 5]. More importantly, CHIKV has the potential to spread globally in the presence of expanding mosquito vector dissemination and increased global travel.

Symptoms of chikungunya fever (CHIKF) include sudden onset of fever, rash, and arthralgia, which predominantly affect the wrist, knee, ankle, and small joints of the hands and feet. Other manifestations include headache, myalgia, joint swelling, and nausea, which have been reported to occur at varying frequencies [1, 2, 6, 7]. Symptoms are generally resolved within 7–10 days [2], but some patients are plagued with chronic arthralgia that could persist for months or years [8, 9].

Disease manifestations of CHIKV infection have been chronicled extensively from different cohorts across various geographic locations [1, 10–12], and common manifestations are similar to those of dengue fever [13]. Given that both viruses share similar geographic distribution and transmission vectors and may cocirculate in many countries [2, 14, 15], it is highly likely that the symptoms of CHIKF are indistinguishable from those of dengue fever during the acute phase of disease. This could lead to a misdiagnosis, especially in rural areas where laboratory testing with polymerase chain reaction (PCR) or serology is limited.

Intensive efforts have been put forward in recent years by various research groups to study how the innate immune response controls CHIKV infection, as well as the CHIKV immunobiological mechanism and pathogenesis [11, 16–19]. In particular, extensive studies have been conducted to characterize the immune mediators profile in patients with CHIKV [20–33]. For example, proinflammatory mediators, such as interleukin 6 (IL-6), monocyte chemoattractant protein 1 (MCP-1), interferon (IFN) α, and IFN-γ, were reported to be elevated during the acute phase of CHIKV infection [25, 27, 32, 33]. Further characterization showed that IL-6 and MCP-1 have been associated with high viral load (HVL) in patients with CHIKF [24, 31], whereas IL-6 and interleukin 1β have been reported to be biomarkers of CHIKF severity [30]. Moreover, IL-6 and granulocyte macrophage colony-stimulating factor were found to be associated with persistent arthralgia [24]. In light of these findings, the challenges of identifying immune signatures specific to CHIKV infection still remain.

In the current study, we attempted to address these complex issues by first examining the disease manifestations reported in a Sri Lankan cohort of 107 patients to determine the combination of disease manifestations that can be used as an indicator for the robust prediction of CHIKV infection. We characterized the circulatory immune mediators profile during the acute phase of infection. After this, we combined the results from the current study with data from all available published studies on acute CHIKV infection from 2009 to 2014, and we conducted a systematic meta-analysis using the pooled data for each immune mediator. The inclusion of various studies for comparison allowed more statistical power for a holistic view of CHIKV-induced immune mediators in the different CHIKV cohorts across various geographic locations. This will help us to identify the immune signatures that better define CHIKV infection.

## MATERIALS AND METHODS

### Patients and Sample Collection

A total of 107 febrile patients were recruited during the 2008 chikungunya outbreak from the Eheliyagoda Divisional Secretariat of Ratnapura District in Sabaragamuwa Province of Sri Lanka. Blood specimens were collected at the government hospital in Eheliyagoda (6°51′0″N, 80°16′0″E) in April 2008, and clinical manifestations were recorded on the day of sampling. Acute-phase specimens were obtained and were confirmed to be CHIKV positive by reverse-transcription PCR (RT-PCR) assay, as described elsewhere [34], and virion-based and peptide-based enzyme-linked immosorbent assay (ELISA) were used to categorize patients into CHIKV (n = 99) and non-CHIKV (n = 8) groups. Non-CHIKV group samples were used as controls in this study. The CHIKV group was further subcategorized according to the RT-PCR results, into a PCR-positive group (PCR value of cycle, <35; n = 71) and a PCR-negative group (PCR value of cycle, >35; n = 28). Using a PCR value of cycle 25 as the threshold, patients in the PCR-positive group patients further classified into HVL (PCR value of cycle <25; n = 46) and low viral load (LVL) (PCR value of cycle, >25; n = 25) groups. Specimens were stored at −80°C until use.

### Study Approval/Ethics Statement

This study was conducted according to Declaration of Helsinki principles, and the use of human samples was approved by the Ethical Review Committee, Faculty of Medicine, University of Kelaniya, Sri Lanka (reference P24/05/2007), with written informed consent obtained from all participants.

### Immunological Studies

CHIKV virion-based and E2EP3-based ELISAs were performed as described elsewhere [35, 36]. Clinical samples are considered anti-CHIKV antibody positive and anti-E2EP3 antibody positive if absorbance values were higher than the mean + 3 standard deviation (SD) values of healthy donor controls.

### Multiplex Microbead Immunoassay for Cytokine Quantification

Plasmatic cytokine levels were measured using the Biosource Human Cytokine Assay Kit (Invitrogen) according to the manufacturer's instructions, as described elsewhere [24]. Results were acquired using the Luminex 200 instrument (Millipore) with IS 2.3 software, based on standard curves plotted through a 5-parameter logistic curve setting.

### Search Strategy, Study Selection, and Data Extraction

PubMed was searched using the search terms *chikungunya, infection,* and *cytokines,* and the search limited to studies assessed on human subjects. Titles, abstracts, full texts, and references of identified studies were screened for citations of further relevant published studies independently by 2 reviewers (T.-S. T. and Y.-W. K.) with differences being resolved through discussion with 2 other reviewers (L. F. P. N. and B. L.). The total data searched and collated for the meta-analysis consisted of 14 distinct studies done in Italy, Gabon, La Reunion, Sri Lanka, India, Thailand, and Singapore [20–33], together with the current Sri Lankan cohort. Actual data were used to compute mean and SD values in the current study and the studies that were published elsewhere [24, 30]. Mean and SD values were extracted directly from the tables provided by 3 of the studies [22, 29, 31]. The median and range values in another study [21] were converted to means and SDs using a method described elsewhere [37]. For 2 other studies [20, 32], the data were extracted from the figures by measuring the pixel positions of the electronic figures and then computing the actual values. For box plots, medians and ranges were used to compute means and SDs using the method as described elsewhere [37], and for scatterplots, the individual values were used to compute means and SDs. Five of the 14 studies were excluded from the meta-analysis because their data could not be extracted for analysis. In 1 study, data were presented as optical density readings and could not be computed into concentration values [26]; 1 study contained data from only a single patient [28]; 2 studies provided only the median or mean of the control group without any estimates of the variance [25, 27]; and 1 study provided median values with interquartile ranges for the patient and control groups [33]. Nevertheless, apart from the single-patient study [26], fold changes were computed for these studies, and they were depicted in the heat maps but not used in the meta-analysis.

### Data Analysis

The data were analyzed after processing using the metafor package in the R statistical language (version 2.15.2). Means and SDs were used to compute standardized mean difference, which was used as the effect size. A random-effects model (DerSimonian-Laird estimator) was used to account for the differences in the studies. Data visualization was done with TIBCO Spotfire (version 5.5) and data processing with Accelry's Pipeline Pilot software (version 9.2). All statistical testing with *P* < .05 was considered to be significant.

### Statistical Analysis

All data are presented as means with standard errors of the mean or SDs. Differences in responses among groups at various time points and between different groups and controls were analyzed using Mann–Whitney *U* tests. Differences in disease manifestations between groups were analyzed using Fisher exact tests. Differences were considered statistically significant at *P* < .05.

## RESULTS

### Clinical Manifestations of Sri Lankan CHIKV Cohort

**Figure 1.**
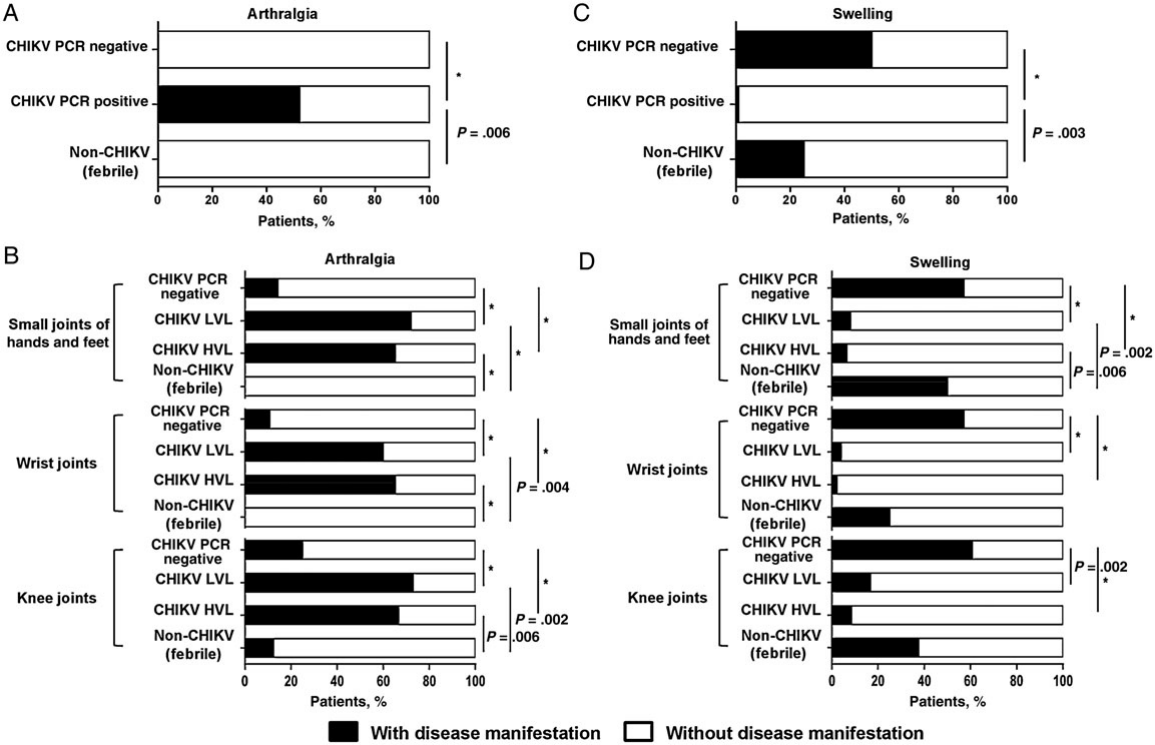
Analysis of chikungunya fever-specific typical disease manifestation from the Sri Lankan patient cohort. Patients were classified as follows: patients with chikungunya virus (CHIKV) infection who were polymerase chain reaction (PCR) positive (positive for viral load detection) (CHIKV PCR positive), patients with CHIKV infection who were PCR negative (negative for viral load detection but positive for CHIKV virion-based or E2EP3-based enzyme-linked immunosorbent assays [ELISAs]) (CHIKV PCR negative), and febrile patients without CHIKV infection (negative both for viral load detection and with CHIKV-based, E2EP3-based ELISAs) (non-CHIKV). *A*, Histograms show the percentage of patients in each group with or without arthralgia in the various joints (a combination of the small joints of hands and feet, wrist joints, and knee joints). *P < .001. *B*, The CHIKV PCR-positive patient group was further segregated into high viral load (HVL) and low viral load (LVL) groups before being compared with the CHIKV PCR-negative and non-CHIKV groups for the presence of arthralgia at the various indicated joints. *P < .001. *C*, Histograms show the percentage of patients in each group with or without swelling in their various joints (small joints of hands and feet, wrist joints, and knee joints). *P < .001. *D*, Patients in the CHIKV PCR-positive group were further segregated into HVL and LVL groups before being compared with the CHIKV PCR-negative and non-CHIKV groups for the presence of swelling at the various indicated joints. Statistical significance was measured using the 2-sided Fisher exact test and indicated the significance of the contingency between 2 categories of patients. *P < .001.

Various studies have reported that CHIKV-infected patients exhibited arthralgia in the wrist, knee, ankle, and small joints of the hands and feet [8, 38]. To determine whether joint-specific arthralgia could be used as a robust marker to identify CHIKV infection, the cohort of Sri Lankan patients was first categorized according to the laboratory parameters (Supplementary Figure 1). These included the use of CHIKV virion-based and E2EP3 epitope-based ELISAs to characterize the anti-CHIKV antibody response [35, 36], as well as RT-PCR to determine whether patients were positive for CHIKV infection [34]. After segregating the patients into non-CHIKV, CHIKV PCR-negative, and CHIKV PCR-positive groups, we next examined the frequency of arthralgia reported by the patients in the small joints of hands and feet, wrist joints, and knee joints. Notably, our analyses revealed that 52% of patients in the CHIKV PCR-positive group had arthralgia in various joints, whereas those in the non-CHIKV group and the febrile patients in the CHIKV PCR-negative group did not (Figure 1*A*). These findings suggested that patients with PCR-positive CHIKV infection had a 50% chance of having arthralgia in all of their joints, and this was statistically significant compared with either the non-CHIKV group or the febrile patients with PCR-negative CHIKV infection (Figure 1*A*).

To further determine whether the CHIKV load had any influence on the occurrence of joint-specific arthralgia, we segregated the CHIKV PCR-positive group into LVL and HVL groups and compared the percentages of patients with arthralgia in their respective indicated joints (Figure 1*B*). Our analyses revealed that a significant majority of patients (>60%) in the PCR-positive CHIKV group exhibited arthralgia in the specific joints, compared with the non-CHIKV or CHIKV PCR-negative groups (Figure 1*B*). Although these results indicated that CHIKV viremia was associated with arthralgia in specific joints, the level of CHIKV load did not correlate with the occurrence of arthralgia (Figure 1*B*).

Next, the patients were characterized based on swelling observed in various joints. Surprisingly, we observed that 99% in the PCR-positive CHIKV group did not exhibit swelling at the various joints examined. In contrast, 25% of patients in the non-CHIKV group and 50% of febrile patients in the PCR-negative CHIKV group exhibited a significant occurrence of joints swelling compared with patients in the PCR-positive CHIKV group (Figure 1*C*). As expected, we also showed that the frequency of swelling in the specific joints was also not affected by viral load (Figure 1*D*).

### Profiles of Immune Mediators in Sri Lankan Patients

**Figure 2.**
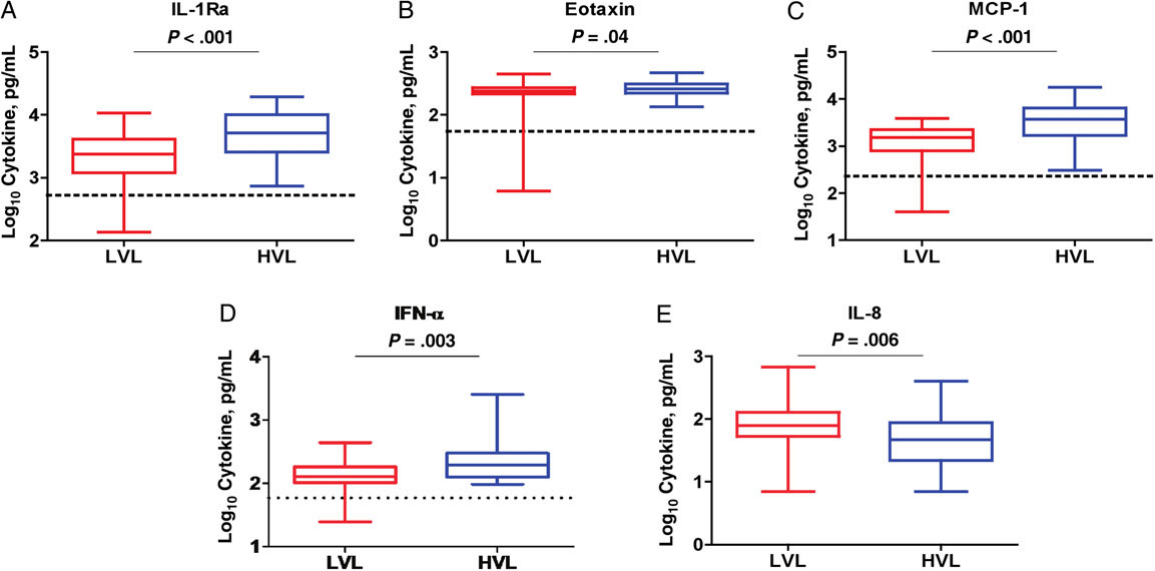
Cytokine and chemokine profiles in patients with polymerase chain reaction (PCR)-positive chikungunya virus (CHIKV) infection. *A–E*, Levels of immune mediators associated with viral load in these patients. Patients with PCR-positive CHIKV infection (n = 71) are classified into 2 groups: high viral load (HVL) and low viral load (LVL). Levels of cytokines and chemokines were compared using 1-tailed Mann–Whitney *U* test. Data represent median levels of mediators detected. Abbreviations: IFN, interferon; IL-1Ra, interleukin 1Ra; IL-8, interleukin 8; MCP-1, monocyte chemoattractant protein 1.

Using a 30-plex microbead immunoassay, we have shown elsewhere that interleukin 1Ra (IL-1Ra), interleukin 2R (IL-2R), interleukin 5, IL-6, interleukin 7 (IL-7), interleukin 8 (IL-8), interleukin 10 (IL-10), interleukin 12 (IL-12), interleukin 15 (IL-15), IFN-α, MCP-1, and interferon gamma-induced protein 10 (IP-10) levels were significantly elevated during the acute phase of CHIKV infection in 2 distinct Singaporean patient cohorts [24, 30]. Hence, we sought to characterize the immune mediator profiles of acute serum samples collected from one of the Sri Lankan cohorts, focusing on the CHIKV PCR-positive group (n = 71) for subsequent analysis. A 2-way hierarchical clustering was performed to provide qualitative analysis of the immune mediator expression profiles from each individual in the HVL and LVL groups (Supplementary Figure 2). We found that levels of IL-1Ra, eotaxin, MCP-1, and IFN-α were significantly elevated in the HVL group compared with the LVL group (Figure 2*A–D*), whereas the level of IL-8 was significantly lower in the HVL group (Figure 2*E*).

### Global Meta-analysis of Immune Mediators in CHIKV Cohorts

The levels of cytokines, chemokines, and growth factors in patients with CHIKV infection have been widely reported from different geographic cohorts. Most studies quantify the levels of immune mediators during the acute phase of infection, and some characterized their levels during the early convalescent to chronic phase [20–32]. In the current study, we sought to collate the data from quantitative studies of immune mediators reported in the different CHIKV cohorts and compared the data with those from the current Sri Lankan patient cohort in this study. A total of 14 published studies were identified (Supplementary Table 1). These studies were based on quantification of immune mediators in either plasma or serum samples from patients with CHIKV infection, using ELISA or bead-based immunoassays. The immune mediators studied included cytokines, chemokines, and growth factors. Supplementary Table 1 summarizes the immune mediators that were significantly increased in CHIKV-infected patients compared with healthy controls.

**Figure 3.**
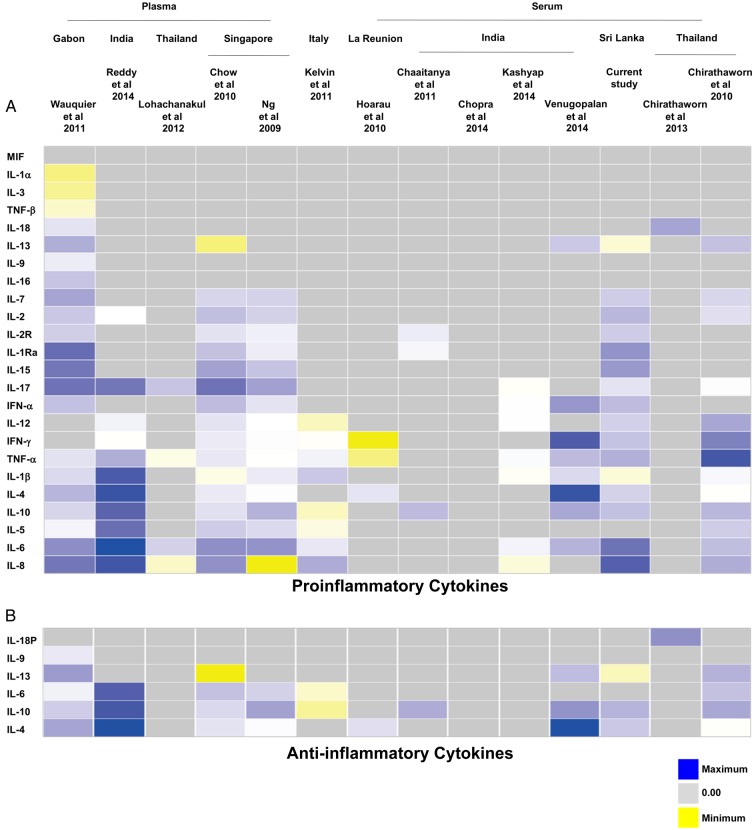
Heat map of immune mediator profiles collected by meta-analysis [20–27, 29–33]. A total of 14 studies were initially searched and collated for the meta-analysis, together with the current Sri Lankan patient cohort. Subsequently, a single-patient study [28] was excluded and the profiles of the total number of immune mediators analyzed were represented by the heat map according to their sample types and geographic locations (in a west-to-east orientation) before being classified as proinflammatory (*A*) or anti-inflammatory (*B*) cytokines. Yellow indicates low expression; blue, high expression; gray, immune mediators not investigated in the studies. Abbreviations: IFN, interferon; IL-1α, interleukin 1α; IL-1β, interleukin 1β; IL-1Ra, interleukin 1Ra; IL-2, interleukin 2; IL-2R, interleukin 2R; IL-3, interleukin 3; IL-4, interleukin 4; IL-5, interleukin 5; IL-6, interleukin 6; IL-7, interleukin 7; IL-8, interleukin 8; IL-9, interleukin 9; IL-10, interleukin 10; IL-12, interleukin 12; IL-13, interleukin 13; IL-15, interleukin 15; IL-16, interleukin 16; IL-17, interleukin 17; IL-18, interleukin 18; IL-18P, interleukin 18P; MIF, macrophage migration inhibitory factor; TNF, tumor necrosis factor.

**Figure 4.**
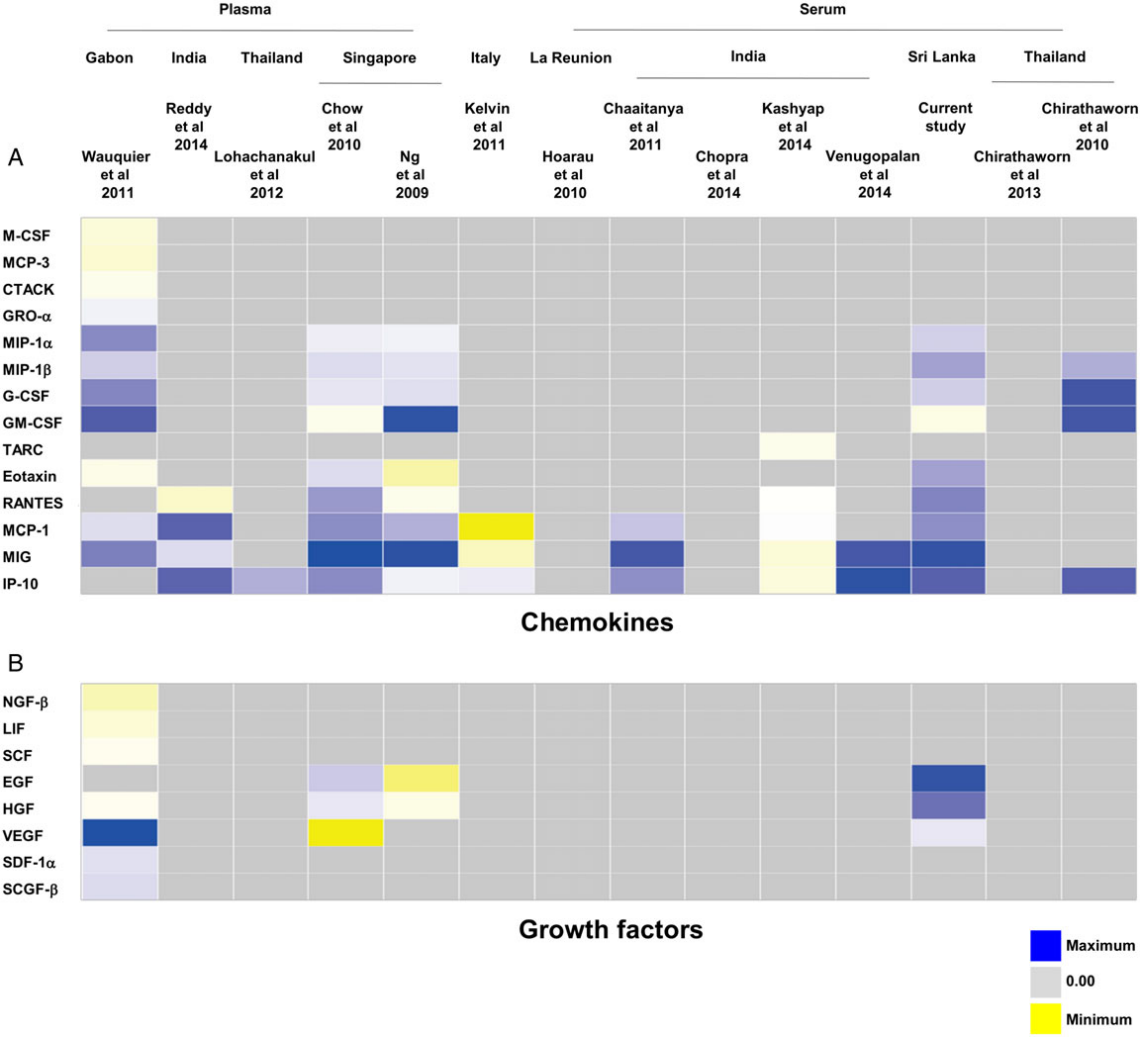
Heat map of immune mediator profiles collected by meta-analysis [20–27, 29– 33]. The profiles of all the immune mediators analyzed in the studies included in Figure 3 were represented by the heat map according to their sample types and geographic locations (in a west-to-east orientation) before being classified as chemokines (*A*) or growth factors (*B*). Each colored well in the 4 heat maps represents the relative levels of each immune mediator. Yellow indicates low expression; blue, high expression; gray, immune mediators not investigated in the studies. Abbreviations: EGF, epidermal growth factor; G-CSF, granulocyte colony-stimulating factor; HGF, hepatocyte growth factor; IP-10, interferon gamma-induced protein 10; MCF, monocyte chemoattractant protein; MIG, monokine induced by gamma interferon; MIP, macrophage inflammatory protein.

Of the 14 studies identified, 5 were excluded from the meta-analysis because their data could not be extracted for analysis: in 1 study, the results from the cytokine assay were presented as optical density readings and could not be computed into concentration values [26]; another study contained data from only a single patient [28]. To overcome the lack of immune mediator profiles from non-CHIKV controls in this study, we used the data from healthy controls in a previously published study [24] as a reference to perform the analysis. A heat map was generated to provide an overall view of the expression profiles of all immune mediators analyzed in the 12 studies, together with data from the current Sri Lankan patient cohort. The immune mediators were segregated according to their functions as proinflammatory cytokines (Figure 3*A*), anti-inflammatory cytokines (Figure 3*B*), chemokines (Figure 4*A*), and growth factors (Figure 4*B*).

**Figure 5.**
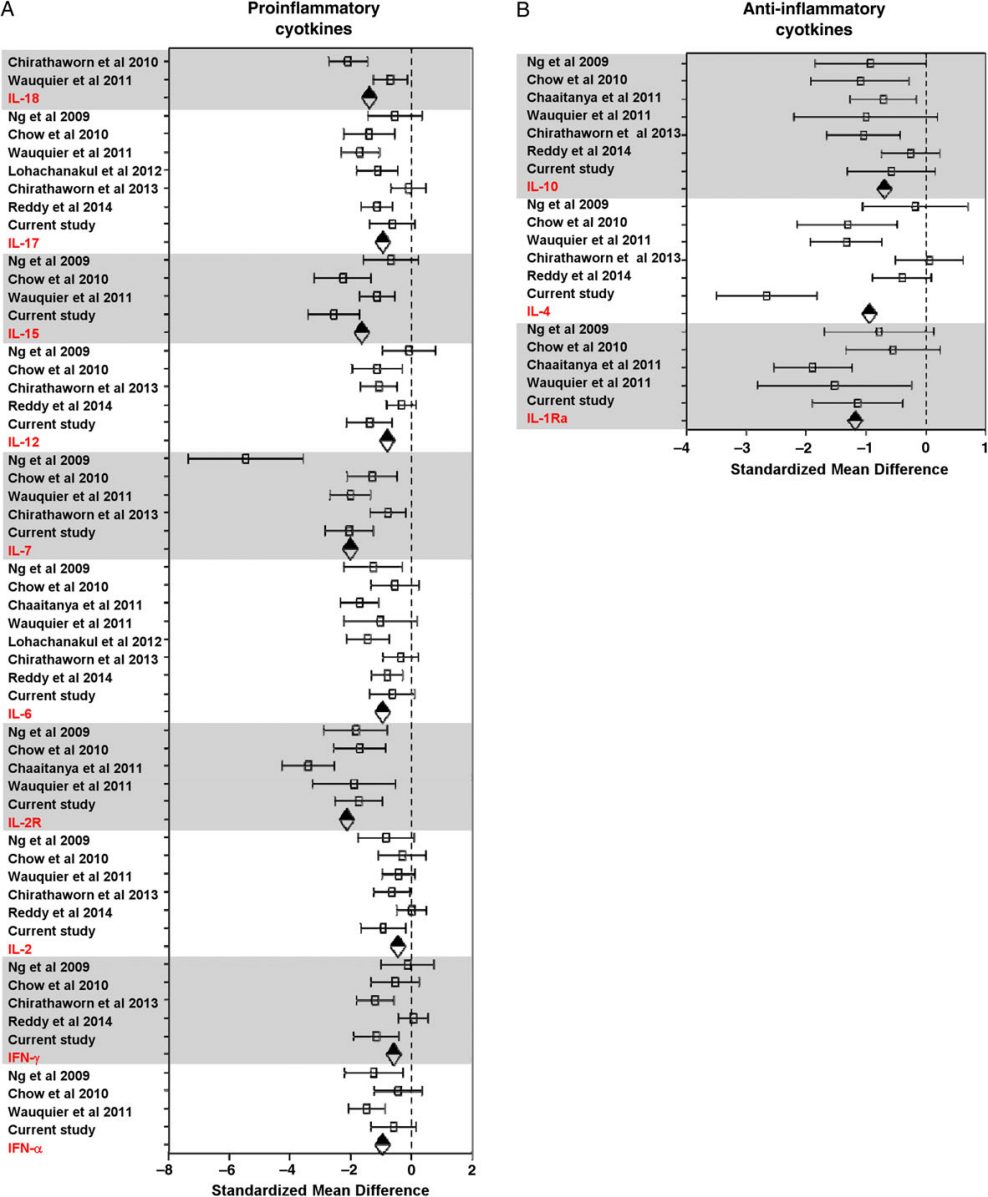
Forest plots of immune mediators that were significantly elevated in different chikungunya virus-infected patient cohorts [20, 21, 24, 29– 32]. *A*, Proinflammatory cytokines. *B*, Anti-inflammatory cytokines. Dotted line represents no difference between mean values for healthy controls and patients with chikungunya fever; diamonds, combined effect size for each immune mediator. Abbreviations: IFN, interferon; IL-1Ra, interleukin 1Ra; IL-2, interleukin 2; IL-2R, interleukin 2R; IL-4, interleukin 4; IL-6, interleukin 6; IL-7, interleukin 7; IL-10, interleukin 10; IL-12, interleukin 12; IL-15, interleukin 15; IL-17, interleukin 17; IL-18, interleukin 18.

**Figure 6.**
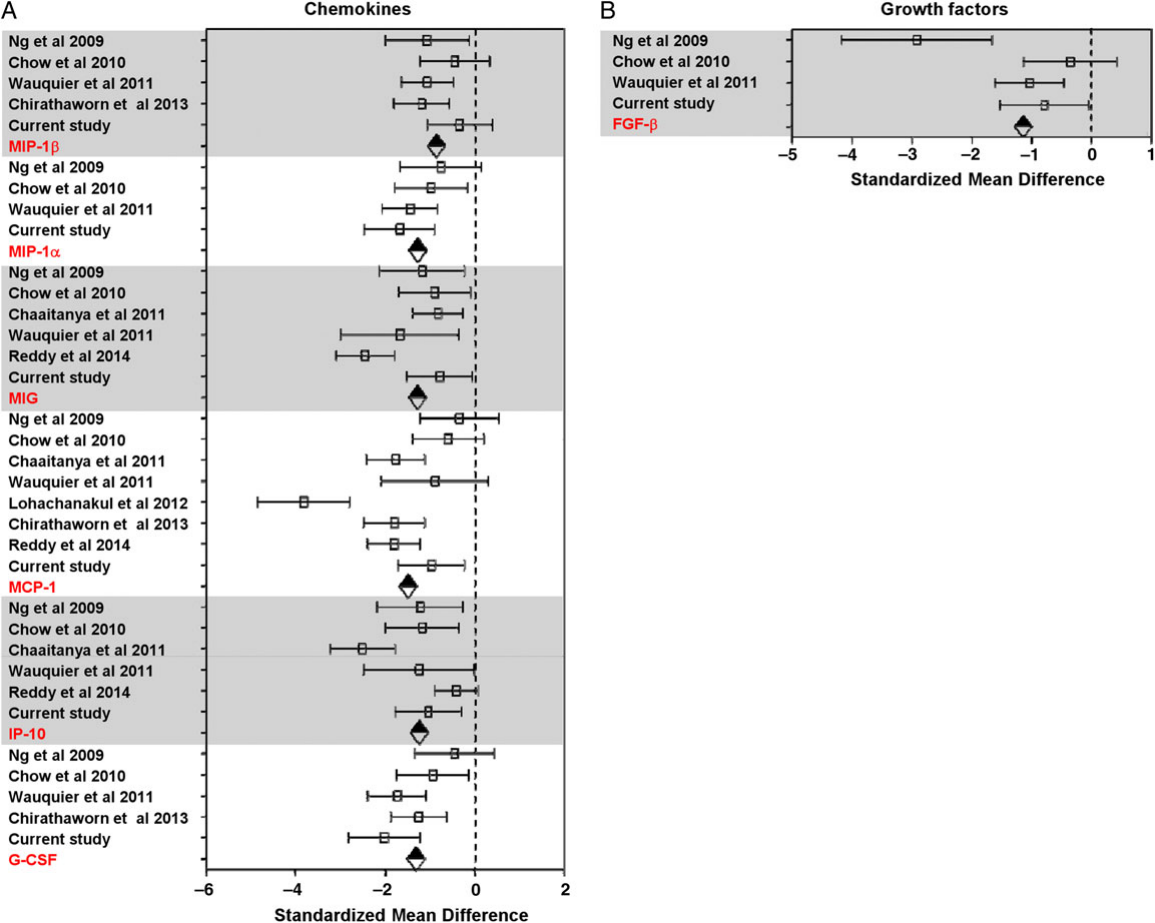
Forest plots of immune mediators that were significantly elevated in different patient cohorts with chikungunya fever (CHIKF) [20, 21, 24, 29–30]. *A*, Chemokines. *B*, Growth factor. Dotted line represents no differences between mean values for healthy controls and patients with chikungunya virus infection; diamonds, combined effect size for each immune mediator. Abbreviations: FGF-β, basic fibroblast growth factor; G-CSF, granulocyte colony-stimulating factor; IP-10, interferon gamma-induced protein 10; MCP-1, monocyte chemoattractant protein 1; MIG, monokine induced by gamma interferon; MIP, macrophage inflammatory protein.

Given that each of the study analyzed different analytes, most of the analytes were present in only a subset of the studies. To gain maximum use of the data, meta-analysis was done on a per-analyte basis using a standard meta-analysis method, as described in “Materials and Methods” section. The pooled data sets of each of the analytes reported in each study were grouped according to the categories of their functions as illustrated in Figures 3 and 4. Using the random-effects model to compare levels of immune mediators, the following levels were significantly elevated in PCR-positive CHIKV-infected patients compared with healthy controls: proinflammatory cytokines (IFN-α, IFN-γ, interleukin 2, IL-2R, IL-6, IL-7, IL-12, IL-15, interleukin 17, and interleukin 18) (Figure 5*A*); anti-inflammatory cytokines IL-1Ra, interleukin 4, and IL-10 (Figure 5*B*); chemokines granulocyte colony-stimulating factor, IP-10, MCP-1, monokine induced by gamma interferon (MIG), macrophage inflammatory protein (MIP) 1α, and MIP-1β (Figure 6*A*); and growth factor basic fibroblast growth factor (FGF-β) (Figure 6*B*). Supplementary Table 2 summarizes results of the random-effects model for the significantly elevated immune mediators in Figure 4. Supplementary Figure 3 shows the forest plots of the proinflammatory cytokines (Supplementary Figure 3*A*), chemokines (Supplementary Figure 3*B*), and growth factors (Supplementary Figure 3*C*) without significant differences; the results from the random-effects models for the nonsignificant immune mediators are summarized in Supplementary Table 3.

## DISCUSSION

CHIKV has gained attention as a clinically important reemerging arbovirus after a quiescent period of almost 50 years [11]. In the last decade, studies have been conducted intensively and extensively to decrypt the immunobiological mechanism and pathogenesis of CHIKV infection. Studies involving patients with CHIKF and the analyses of clinical samples provided the most valuable information in terms of identifying the disease manifestations that define CHIKF and understanding the immune control of CHIKV infection in humans.

It is well documented that arthralgia is a hallmark of CHIKV infection during the acute and chronic phases of the disease [8, 39, 40]. Here, we show that arthralgia at the wrist joints, knee joints, and small joints of both hands and feet were significant indicators of CHIKV infection during the acute phase of disease when we compared CHIKV-infected PCR-positive patients with non–CHIKV-infected patients or febrile patients who were PCR negative but serologically positive. To our knowledge, this is the first study showing a significant association of CHIKV load with arthralgia as an indicator of acute CHIKV infection. Together, these findings strongly indicate that joint-specific arthralgia is a good indicator of CHIKV infection, because earlier studies have suggested that arthralgia is predictive of CHIKV infection but not dengue virus (DENV) infection [38]. However, when the Sri Lankan patient cohort was examined for the frequencies of swelling at the wrist joints, knee joints, and small joints of hands and feet, significant associations were not observed.

The cocirculation of multiple pathogens that result in similar disease manifestations in infected individuals within the same geographic area could complicate the accuracy of clinical diagnosis [15]. For example, India was hit by an outbreak of Kyasanur Forest disease, a tick-borne hemorrhagic fever caused by Kyasanur Forest disease virus [41]. The disease manifestations of Kyasanur Forest disease overlap with those of CHIKF. Therefore, there is a need to define the disease manifestations specific to a particular disease (eg, CHIKV infection) when multiple pathogens are prevalent in the same geographic area.

Multiple studies have been conducted to clarify and characterize the CHIKF disease manifestations and immune mediator profiles during different phases of the disease across different geographic locations [20–32]. However, results varied between studies, which used different preparations of the clinical specimens and different assay platforms. For example, the type of anticoagulant (ethylenediaminetetraacetic acid, sodium citrate dextrose, or sodium heparin) used for either plasma or serum collections and the type of detection kits used (ELISA or multiplex microbead immunoassay, from different manufacturers) differed widely between studies. Such differences will lead to variations in the results obtained from the analyses of circulatory immune mediators [42–44].

Nevertheless, we found that a set of immune mediators showed significant differences in their levels in CHIKV-infected PCR-positive patients when compared with non–CHIKV-infected or CHIKV-infected PCR-negative febrile patients (Supplementary Figure 2). These results were similar to those from an earlier study in a Singapore cohort of 30 patients [24], wherein IL-1Ra, IL-6, IL-7, IL-8, IL-12, IL-15, IFN-α, MCP-1, and IP-10 were shown to be significantly induced during acute infection. Levels of IL-2R, interleukin 4, MIP-1α, MIP-1β, MIG, RANTES (regulated on activation, normal T-expressed, and presumably secreted), eotaxin, granulocyte colony-stimulating factor, epidermal growth factor, FGF-β, and hepatocyte growth factor were reported to be significantly higher after the acute phase. The differences in these findings could reflect differences in the patient samples used for analysis; serum samples were used in the current study, and plasma samples in the previous studies [24, 30].

IFN-α is a critical mediator of anti-CHIKV immune response [18, 45]. Here, we established that a higher level of IFN-α was found to be associated with a higher viral load, which was in agreement with the previous observation that the level of cytokines was dependent on the viral load to mediate a stronger anti-CHIKV response [24]. In addition, cytokines and chemokines (IL-1Ra, MCP-1, and eotaxin) were found to be associated with the level of viral load in the Sri Lankan patient cohort. Furthermore, the roles of monocytes and macrophages have been implicated in CHIKV pathogenesis [46–48]. Hence, it is no surprise that the level of MCP-1, the major chemoattractant for monocytes and macrophages, was consistently reported to be significantly higher in patients with CHIKF from numerous studies [20, 21, 24, 26, 29, 31, 32], as well as being associated with the level of CHIKV load [24, 31].

**Figure 7.**
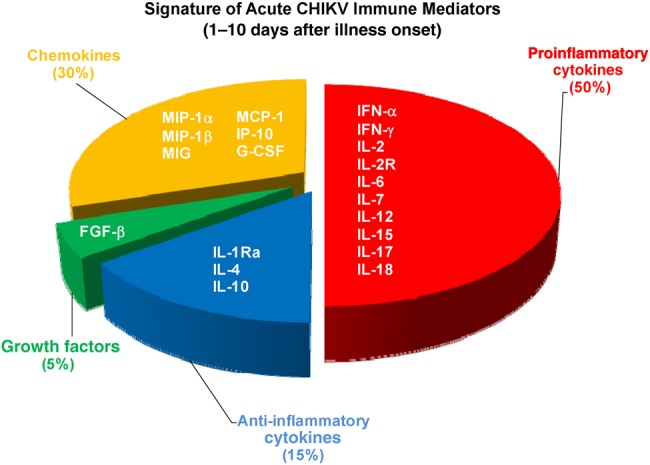
Signature of immune mediators in patients with acute chikungunya virus (CHIKV) infection. Immune mediators were categorized into proinflammatory cytokines (*red*), anti-inflammatory cytokines (*blue*), growth factors (*green*), and chemokines (*yellow*). Pie chart shows percentage of elevated immune mediators from each subcategory. Abbreviations: FGF-β, basic fibroblast growth factor; G-CSF, granulocyte colony-stimulating factor; IFN, interferon; IL-1Ra, interleukin 1Ra; IL-2, interleukin 2; IL-2R, interleukin 2R; IL-4, interleukin 4; IL-6, interleukin 6; IL-7, interleukin 7; IL-10, interleukin 10; IL-12, interleukin 12; IL-15, interleukin 15; IL-17, interleukin 17; IL-18, interleukin 18; IP-10, interferon gamma-induced protein 10; MCP-1, monocyte chemoattractant protein 1; MIG, monokine induced by gamma interferon; MIP, macrophage inflammatory protein.

The meta-analysis revealed an elevation of multiple statistically significant immune mediators in patients with CHIKF during the acute phase of disease, compared with healthy controls in multiple independent cohorts from different geographic locations (Figures 5 and 6). Thus, a combination of cytokine and chemokine immune signatures can be used in tandem to act as robust biomarkers that positively predict CHIKV infection (Figure 7). Owing to the cocirculation of multiple pathogens within the same geographic area [15], distinguishing biomarker signatures between different viral infections, such as CHIKV and DV, will be important to improve the accuracy of clinical prognosis during the early stage of disease. Preliminary systematic and meta-analysis reveals that DENV induces a set of biomarkers that overlaps with the acute CHIKV immune mediators presented in this study: IFN-γ, IL-6, IL-10, MIP-1β, and MCP-1 (Supplementary Table 4). However, specific biomarkers for the 2 viral infections could also be distinguished (TNF-α for DENV and IFN-α for CHIKV). To date, we know of no study/review that has systematically summarized the immune mediator profiles of different DENV cohorts across different geographic locations, and detailed systematic and meta-analysis across multiple DENV cohorts will provide a basis for further refinements in clinical prognosis and management.

Although the studies on CHIKV-induced immune mediators have been limited to adult patients so far, it is imperative to characterize the immune response of CHIKF in pediatric cohorts for comparison and to identify any age differences. The age of patients with CHIKF has been shown elsewhere to be one of the factors associated with relapsing or lingering rheumatic musculoskeletal pain [9, 49]. It would also be interesting to understand the relationship between age and the expression of immune genes in CHIKV infection, as demonstrated in mouse models [45].

To our knowledge, our systematic review and meta-analysis is the first to examine the immune mediator profiles of different CHIKF cohorts during the 2008–2010 outbreaks across different geographic locations. The identification of multiple statistically significant immune signatures from these independent cohorts will provide a better basis for establishing a set of biomarkers that define acute CHIKV infection. Further validation in new CHIKF patient cohorts, such as those in the Americas, would be valuable to ascertain the robustness of the set of biomarkers for differentiating CHIKV-infected individuals from those without CHIKV infection.

## Supplementary Data

Supplementary materials are available at *The Journal of Infectious Diseases* online (http://jid.oxfordjournals.org). Supplementary materials consist of data provided by the author that are published to benefit the reader. The posted materials are not copyedited. The contents of all supplementary data are the sole responsibility of the authors. Questions or messages regarding errors should be addressed to the author.

Supplementary Data
