# Supplementary material for: A Systematic Meta-analysis of Immune Signatures in Patients With Acute Chikungunya Virus Infection
Source: J Infect Dis. 2015 Jan 29;211(12):1925–35. doi: 10.1093/infdis/jiv049 (PMC4442625; doi:10.1093/infdis/jiv049)
Supplement: Supplementary Data [file supp_211_12_1925__index.html]

A Systematic Meta-analysis of Immune Signatures in Patients With Acute Chikungunya Virus Infection — A Systematic Meta-analysis of Immune Signatures in Patients With Acute Chikungunya Virus Infection — Supplementary Data 

# A Systematic Meta-analysis of Immune Signatures in Patients With Acute Chikungunya Virus Infection

## Supplementary Data

Supplementary Data

**Files in this Data Supplement:**

- Supplementary Data - Doc file
- Supplementary Tables - doc file
