# Supplementary material for: A Systematic Meta-analysis of Immune Signatures in Patients With Acute Chikungunya Virus Infection
Source: J Infect Dis. 2015 Jan 29;211(12):1925–35. doi: 10.1093/infdis/jiv049 (PMC4442625; doi:10.1093/infdis/jiv049)

**Supplementary Data**

**Supplementary Table 1.** Expression profiles of circulatory immune mediators in CHIKV-infected patients from 14 independent published cohorts and the current Sri Lankan patient cohort. The cytokines, chemokines and growth factors that were significantly elevated during the acute phase of the infection when compared to controls are presented in the table.

| **Origin** |  | **Year** |  | **Immune mediators** | |  | **Reference** |
| --- | --- | --- | --- | --- | --- | --- | --- |
| Hong Kong |  | 2006 |  | IL-8 | MCP-1 |  | (28) |
|  |  |  |  | MIG | IP-10 |  |  |
|  |  |  |  |  |  |  |  |
|  |  |  |  |  |  |  |  |
| La Reunion |  | 2006-2007 |  | IL-12 | IFN-γ  |  | (25) |
|  |  |  |  |  |  |  |  |
|  |  |  |  |  |  |  |  |
| Gabon |  | 2007 |  | IL-1Ra | IFN-γ  |  | (26) |
|  |  |  |  | IL-2Ra | MCP-1 |  |  |
|  |  |  |  | IL-4 | MIP-1β |  |  |
|  |  |  |  | IL-6 | CXCL12 |  |  |
|  |  |  |  | IL-7 | MIF |  |  |
|  |  |  |  | IL-8 | IP-10 |  |  |
|  |  |  |  | IL-12p40 | G-CSF |  |  |
|  |  |  |  | IL-13 | GM-CSF |  |  |
|  |  |  |  | IL-16 | VEGF |  |  |
|  |  |  |  | IL-17 | SCGF-β |  |  |
|  |  |  |  | IFN-α | PDGF-β |  |  |
|  |  |  |  |  |  |  |  |
|  |  |  |  |  |  |  |  |
| Italy |  | 2007 |  | IL-6 | MIG |  | (27) |
|  |  |  |  | MCP-1 | IP-10 |  |  |
|  |  |  |  |  |  |  |  |
|  |  |  |  |  |  |  |  |

| **Supplementary Table 1.** (continued) | | | | | | | |
| --- | --- | --- | --- | --- | --- | --- | --- |
|  |  |  |  |  |  |  |  |
| **Origin** |  | **Year** |  | **Immune mediators** | |  | **Reference** |
| India |  | 2006 |  | IL-1 β | IFN-α |  | (23) |
|  |  | 2006 |  | IL-4 | IFN-β |  | (26) |
|  |  | 2006 |  | IL-6 | IFN-γ |  | (24) |
|  |  | 2008 |  | IL-8 | MCP-1 |  | (20) |
|  |  | 2010 |  | IL-10 | MIG |  | (31) |
|  |  |  |  | IL-13 | IP-10 |  |  |
|  |  |  |  | IL-17 | RANTES |  |  |
|  |  |  |  |  | TARC |  |  |
|  |  |  |  |  |  |  |  |
|  |  |  |  |  |  |  |  |
| Thailand |  | 2008 |  | IL-6 | MCP-1 |  | (21) |
|  |  | 2008 |  | IL-8 | G-CSF |  | (22) |
|  |  | 2009-2010 |  | IL-18 | GM-CSF |  | (29) |
|  |  |  |  | IL-18BP | TNF-α |  |  |
|  |  |  |  |  |  |  |  |
|  |  |  |  |  |  |  |  |
| Singapore |  | 2008 |  | IL-1RA | IL-10 |  | (30) |
|  |  | 2009 |  | IL-2R | IL-12p40 |  | (24) |
|  |  |  |  | IL-5 | IL-15 |  |  |
|  |  |  |  | IL-6 | IFN-α |  |  |
|  |  |  |  | IL-7 | MCP-1 |  |  |
|  |  |  |  | IL-8 | IP-10 |  |  |
|  |  |  |  |  |  |  |  |
|  |  |  |  |  |  |  |  |
| Sri Lanka |  | 2008 |  | IL-1RA | MIP-1β |  | Current |
|  |  |  |  | IL-2R | MIG |  | study |
|  |  |  |  | IL-4 | IP-10 |  |  |
|  |  |  |  | IL-6 | RANTES |  |  |
|  |  |  |  | IL-7 | Eotaxin |  |  |
|  |  |  |  | IL-8 | G-CSF |  |  |
|  |  |  |  | IL-12p40 | EGF |  |  |
|  |  |  |  | IL-15 | VEGF |  |  |
|  |  |  |  | IFN-α | FGF-basic |  |  |
|  |  |  |  | MCP-1 | HGF |  |  |
|  |  |  |  | MIP-1 α |  |  |  |
|  |  |  |  |  |  |  |  |
|  |  |  |  |  |  |  |  |

**Supplementary Table 2.** Results of random effects models for the significant immune mediators in the meta-analysis. Column YI shows the standardized mean difference. The 95% confidence intervals for the standardized mean differences are also provided in the columns “CI lower” and “CI upper”. The meta-analysis P value is provided for each mediator together with the I^2^ (a quantity for consistency as described in [49]) and the P value of the heterogeneity test.

| **Study** | **Cohort location** | **Sample type** | **Analyte** | **YI** | **CI lower** | **CI upper** |
| --- | --- | --- | --- | --- | --- | --- |
| Ng et al 2009 | Singapore | Plasma | IFN-α | -1.24 | -2.20 | -0.29 |
| Chow et al 2010 | Singapore | Plasma | IFN-α | -0.45 | -1.24 | 0.34 |
| Wauquier et al 2011 | Gabon | Plasma | IFN-α | -1.48 | -2.09 | -0.87 |
| Current study | Sri Lanka | Serum | IFN-α | -0.60 | -1.34 | 0.14 |
| IFN-α (p value = 3.10E-04) | |  | IFN-α | -0.96 | -1.48 | -0.44 |
| I^2^ = 46.77; heterogeneity p value = 1.31E-01 | | | | | | |
| Ng et al 2009 | Singapore | Plasma | IFN-γ | -0.14 | -1.01 | 0.74 |
| Chow et al 2010 | Singapore | Plasma | IFN-γ | -0.54 | -1.33 | 0.25 |
| Chirathaworn et al 2013 | Thailand | Serum | IFN-γ | -1.20 | -1.82 | -0.58 |
| Reddy et al 2014 | India | Plasma | IFN-γ | 0.05 | -0.44 | 0.54 |
| Current study | Sri Lanka | Serum | IFN-γ | -1.17 | -1.92 | -0.41 |
| IFN-γ (p value = 3.92E-02) | |  | IFN-γ | -0.59 | -1.15 | -0.03 |
| I^2^ = 70.17; heterogeneity p value = 9.43E-03 | | | | | | |
| Ng et al 2009 | Singapore | Plasma | IL-12 | -0.08 | -0.96 | 0.79 |
| Chow et al 2010 | Singapore | Plasma | IL-12 | -1.15 | -1.97 | -0.32 |
| Chirathaworn et al 2013 | Thailand | Serum | IL-12 | -1.08 | -1.70 | -0.47 |
| Reddy et al 2014 | India | Plasma | IL-12 | -0.34 | -0.83 | 0.15 |
| Current study | Sri Lanka | Serum | IL-12 | -1.39 | -2.15 | -0.63 |
| IL-12 (p value = 1.11E-03) | |  | IL-12 | -0.80 | -1.28 | -0.32 |
| I^2^ = 58.51; heterogeneity p value = 4.69E-02 | | | | | | |
| Ng et al 2009 | Singapore | Plasma | IL-15 | -0.69 | -1.59 | 0.22 |
| Chow et al 2010 | Singapore | Plasma | IL-15 | -2.27 | -3.20 | -1.34 |
| Wauquier et al 2011 | Gabon | Plasma | IL-15 | -1.14 | -1.73 | -0.56 |
| Current study | Sri Lanka | Serum | IL-15 | -2.56 | -3.40 | -1.73 |
| IL-15 (p value = 1.30E-04) | |  | IL-15 | -1.65 | -2.49 | -0.80 |

**Supplementary Table 2.** (continued)

| Ng et al 2009 | Singapore | Plasma | IL-17 | -0.56 | -1.45 | 0.34 |
| --- | --- | --- | --- | --- | --- | --- |
| Chow et al 2010 | Singapore | Plasma | IL-17 | -1.40 | -2.24 | -0.56 |
| Wauquier et al 2011 | Gabon | Plasma | IL-17 | -1.70 | -2.34 | -1.07 |
| Lohachanakul et al 2012 | Thailand | Plasma | IL-17 | -1.12 | -1.80 | -0.45 |
| Chirathaworn et al 2013 | Thailand | Serum | IL-17 | -0.09 | -0.67 | 0.48 |
| Reddy et al 2014 | India | Plasma | IL-17 | -1.14 | -1.67 | -0.62 |
| Current study | Sri Lanka | Serum | IL-17 | -0.64 | -1.38 | 0.10 |
| IL-17 (p value = 1.77E-05) | |  | IL-17 | -0.95 | -1.39 | -0.52 |
| I^2^ = 65.23; heterogeneity p value = 8.39E-03 | | | | | | |
| Chirathaworn et al 2010 | Thailand | Serum | IL-18 | -2.10 | -2.73 | -1.46 |
| Wauquier et al 2011 | Gabon | Plasma | IL-18 | -0.71 | -1.27 | -0.15 |
| IL-18 (p value = 4.49E-02) | |  | IL-18 | -1.40 | -2.76 | -0.03 |
| I^2^ = 90.36; heterogeneity p value = 1.28E-03 | | | | | | |
| Ng et al 2009 | Singapore | Plasma | IL-2 | -0.84 | -1.76 | 0.07 |
| Chow et al 2010 | Singapore | Plasma | IL-2 | -0.30 | -1.09 | 0.48 |
| Wauquier et al 2011 | Gabon | Plasma | IL-2 | -0.43 | -0.98 | 0.11 |
| Chirathaworn et al 2013 | Thailand | Serum | IL-2 | -0.66 | -1.25 | -0.07 |
| Reddy et al 2014 | India | Plasma | IL-2 | 0.00 | -0.49 | 0.49 |
| Current study | Sri Lanka | Serum | IL-2 | -0.93 | -1.68 | -0.19 |
| IL-2 (p value = 2.38E-03) | |  | IL-2 | -0.45 | -0.75 | -0.16 |
| I^2^ = 19.36; heterogeneity p value = 2.87E-01 | | | | | | |
| Ng et al 2009 | Singapore | Plasma | IL-2R | -1.84 | -2.89 | -0.80 |
| Chow et al 2010 | Singapore | Plasma | IL-2R | -1.70 | -2.57 | -0.84 |
| Chaaitanya et al 2011 | India | Serum | IL-2R | -1.89 | -3.25 | -0.53 |
| Wauquier et al 2011 | Gabon | Plasma | IL-2R | -3.40 | -4.25 | -2.56 |
| Current study | Sri Lanka | Serum | IL-2R | -1.75 | -2.53 | -0.97 |
| IL-2R (p value = 2.13E-09) | |  | IL-2R | -2.14 | -2.84 | -1.44 |
| I^2^ = 63.26; heterogeneity p value = 2.79E-02 | | | | | | |
| Ng et al 2009 | Singapore | Plasma | IL-6 | -1.27 | -2.23 | -0.31 |
| Chow et al 2010 | Singapore | Plasma | IL-6 | -0.55 | -1.34 | 0.24 |
| Chaaitanya et al 2011 | India | Serum | IL-6 | -1.03 | -2.23 | 0.18 |
| Wauquier et al 2011 | Gabon | Plasma | IL-6 | -1.71 | -2.35 | -1.08 |
| Lohachanakul et al 2012 | Thailand | Plasma | IL-6 | -1.45 | -2.15 | -0.75 |
| Chirathaworn et al 2013 | Thailand | Serum | IL-6 | -0.36 | -0.94 | 0.22 |
| Reddy et al 2014 | India | Plasma | IL-6 | -0.80 | -1.31 | -0.30 |
| Current study | Sri Lanka | Serum | IL-6 | -0.64 | -1.38 | 0.09 |
| IL-6 (p value = 1.80E-07) | |  | IL-6 | -0.96 | -1.32 | -0.60 |
| I^2^ = 49.89; heterogeneity p value = 5.17E-02 | | | | | | |

**Supplementary Table 2.** (continued)

| Ng et al 2009 | Singapore | Plasma | IL-7 | -5.45 | -7.36 | -3.55 |
| --- | --- | --- | --- | --- | --- | --- |
| Chow et al 2010 | Singapore | Plasma | IL-7 | -1.31 | -2.14 | -0.47 |
| Wauquier et al 2011 | Gabon | Plasma | IL-7 | -2.02 | -2.69 | -1.36 |
| Chirathaworn et al 2013 | Thailand | Serum | IL-7 | -0.78 | -1.37 | -0.19 |
| Current study | Sri Lanka | Serum | IL-7 | -2.06 | -2.86 | -1.27 |
| IL-7 (p value = 2.80E-05) | |  | IL-7 | -2.02 | -2.97 | -1.08 |
| I^2^ = 84.81; heterogeneity p value = 2.70E-05 | | | | | | |
| Ng et al 2009 | Singapore | Plasma | IL-1Ra | -0.78 | -1.69 | 0.13 |
| Chow et al 2010 | Singapore | Plasma | IL-1Ra | -0.55 | -1.34 | 0.24 |
| Chaaitanya et al 2011 | India | Serum | IL-1Ra | -1.53 | -2.81 | -0.24 |
| Wauquier et al 2011 | Gabon | Plasma | IL-1Ra | -1.89 | -2.54 | -1.24 |
| Current study | Sri Lanka | Serum | IL-1Ra | -1.14 | -1.89 | -0.38 |
| IL-1Ra (p value = 1.26E-05) | |  | IL-1Ra | -1.18 | -1.71 | -0.65 |
| I^2^ = 49.83; heterogeneity p value = 9.26E-02 | | | | | | |
| Ng et al 2009 | Singapore | Plasma | IL-4 | -0.18 | -1.06 | 0.70 |
| Chow et al 2010 | Singapore | Plasma | IL-4 | -1.31 | -2.14 | -0.48 |
| Wauquier et al 2011 | Gabon | Plasma | IL-4 | -1.33 | -1.93 | -0.73 |
| Chirathaworn et al 2013 | Thailand | Serum | IL-4 | 0.06 | -0.51 | 0.63 |
| Reddy et al 2014 | India | Plasma | IL-4 | -0.40 | -0.89 | 0.09 |
| Current study | Sri Lanka | Serum | IL-4 | -2.66 | -3.50 | -1.82 |
| IL-4 (p value = 1.21E-02) | |  | IL-4 | -0.95 | -1.68 | -0.21 |
| I^2^ = 86.22; heterogeneity p value = 8.33E-07 | | | | | | |
| Ng et al 2009 | Singapore | Plasma | IL-10 | -0.93 | -1.85 | 0.00 |
| Chow et al 2010 | Singapore | Plasma | IL-10 | -1.10 | -1.92 | -0.28 |
| Chaaitanya et al 2011 | India | Serum | IL-10 | -1.00 | -2.20 | 0.20 |
| Wauquier et al 2011 | Gabon | Plasma | IL-10 | -0.71 | -1.27 | -0.15 |
| Chirathaworn et al 2013 | Thailand | Serum | IL-10 | -1.04 | -1.65 | -0.43 |
| Reddy et al 2014 | India | Plasma | IL-10 | -0.25 | -0.74 | 0.23 |
| Current study | Sri Lanka | Serum | IL-10 | -0.57 | -1.31 | 0.16 |
| IL-10 (p value = 1.11E-07) | |  | IL-10 | -0.69 | -0.95 | -0.44 |
| I^2^ = 0.00; heterogeneity p value = 4.32E-01 | | | | | | |
| Ng et al 2009 | Singapore | Plasma | G-CSF | -0.46 | -1.35 | 0.43 |
| Chow et al 2010 | Singapore | Plasma | G-CSF | -0.94 | -1.75 | -0.14 |
| Wauquier et al 2011 | Gabon | Plasma | G-CSF | -1.74 | -2.38 | -1.10 |
| Chirathaworn et al 2013 | Thailand | Serum | G-CSF | -1.26 | -1.88 | -0.63 |
| Current study | Sri Lanka | Serum | G-CSF | -2.03 | -2.82 | -1.23 |
| G-CSF (p value = 2.51E-07 ) | | | G-CSF | -1.32 | -1.82 | -0.82 |
| I^2^ = 56.31; heterogeneity p value = 5.73E-02 | | | | | | |

**Supplementary Table 2.** (continued)

| Ng et al 2009 | Singapore | Plasma | IP-10 | -1.22 | -2.18 | -0.27 |
| --- | --- | --- | --- | --- | --- | --- |
| Chow et al 2010 | Singapore | Plasma | IP-10 | -1.19 | -2.01 | -0.36 |
| Chaaitanya et al 2011 | India | Serum | IP-10 | -1.24 | -2.48 | -0.01 |
| Wauquier et al 2011 | Gabon | Plasma | IP-10 | -2.51 | -3.23 | -1.79 |
| Reddy et al 2014 | India | Plasma | IP-10 | -0.42 | -0.91 | 0.07 |
| Current study | Sri Lanka | Serum | IP-10 | -1.04 | -1.79 | -0.29 |
| IP-10 (p value = 2.43E-04) | | | IP-10 | -1.25 | -1.92 | -0.58 |
| I^2^ = 77.44; heterogeneity p value = 4.87E-04 | | | | | | |
| Ng et al 2009 | Singapore | Plasma | MCP-1 | -0.35 | -1.23 | 0.54 |
| Chow et al 2010 | Singapore | Plasma | MCP-1 | -0.60 | -1.39 | 0.20 |
| Chaaitanya et al 2011 | India | Serum | MCP-1 | -0.89 | -2.08 | 0.30 |
| Wauquier et al 2011 | Gabon | Plasma | MCP-1 | -1.77 | -2.41 | -1.13 |
| Lohachanakul et al 2012 | Thailand | Plasma | MCP-1 | -3.82 | -4.86 | -2.79 |
| Chirathaworn et al 2013 | Thailand | Serum | MCP-1 | -1.80 | -2.48 | -1.13 |
| Reddy et al 2014 | India | Plasma | MCP-1 | -1.81 | -2.38 | -1.23 |
| Current study | Sri Lanka | Serum | MCP-1 | -0.97 | -1.72 | -0.23 |
| MCP-1 (p value = 3.01E-06 ) | | | MCP-1 | -1.49 | -2.12 | -0.87 |
| I^2^ =80.81 ; heterogeneity p value = 5.89E-06 | | | | | | |
| Ng et al 2009 | Singapore | Plasma | MIG | -1.18 | -2.13 | -0.23 |
| Chow et al 2010 | Singapore | Plasma | MIG | -0.89 | -1.70 | -0.09 |
| Chaaitanya et al 2011 | India | Serum | MIG | -1.68 | -2.99 | -0.36 |
| Wauquier et al 2011 | Gabon | Plasma | MIG | -0.83 | -1.39 | -0.26 |
| Reddy et al 2014 | India | Plasma | MIG | -2.45 | -3.09 | -1.81 |
| Current study | Sri Lanka | Serum | MIG | -0.79 | -1.53 | -0.05 |
| MIG (p value = 3.66E-05) | |  | MIG | -1.29 | -1.90 | -0.68 |
| I^2^ = 72.55; heterogeneity p value =2.69E-03 | | | | | | |
| Ng et al 2009 | Singapore | Plasma | MIP-1α | -0.76 | -1.67 | 0.15 |
| Chow et al 2010 | Singapore | Plasma | MIP-1α | -0.99 | -1.80 | -0.17 |
| Wauquier et al 2011 | Gabon | Plasma | MIP-1α | -1.45 | -2.06 | -0.84 |
| Current study | Sri Lanka | Serum | MIP-1α | -1.68 | -2.46 | -0.91 |
| MIP-1α (p value = 4.06E-11) | |  | MIP-1α | -1.28 | -1.67 | -0.90 |
| I^2^ = 2.62; heterogeneity p value = 3.79E-01 | | | | | | |
| Ng et al 2009 | Singapore | Plasma | MIP-1β | -1.07 | -2.01 | -0.13 |
| Chow et al 2010 | Singapore | Plasma | MIP-1β | -0.46 | -1.24 | 0.33 |
| Wauquier et al 2011 | Gabon | Plasma | MIP-1β | -1.07 | -1.65 | -0.49 |
| Chirathaworn et al 2013 | Thailand | Serum | MIP-1β | -1.20 | -1.82 | -0.58 |
| Current study | Sri Lanka | Serum | MIP-1β | -0.35 | -1.08 | 0.39 |
| MIP-1β (p value = 9.05E-07) | |  | MIP-1β | -0.86 | -1.21 | -0.52 |
| I^2^ = 15.73; heterogeneity p value = 3.14E-01 | | | | | | |

**Supplementary Table 2.** (continued)

| Ng et al 2009 | Singapore | Plasma | FGF-β | -2.91 | -4.17 | -1.66 |
| --- | --- | --- | --- | --- | --- | --- |
| Chow et al 2010 | Singapore | Plasma | FGF-β | -0.35 | -1.14 | 0.43 |
| Wauquier et al 2011 | Gabon | Plasma | FGF-β | -1.03 | -1.61 | -0.46 |
| Current study | Sri Lanka | Serum | FGF-β | -0.79 | -1.53 | -0.05 |
| FGF-β (p value = 4.72E-03) | |  | FGF-β | -1.14 | -1.93 | -0.35 |
| I^2^ = 74.65; heterogeneity p value = 7.96E-03 | | | | | | |

**Supplementary Table 3.** Results of random effects models for the non-significant immune mediators in the meta-analysis. Column YI shows the standardized mean difference. The 95% confidence intervals for the standardized mean differences are also provided in the columns “CI lower” and “CI upper”. The meta-analysis P value is provided for each mediator together with the I^2^ (a quantity for consistency as described in [49]) and the P value of the heterogeneity test.

| **Study** | **Cohort location** | **Sample type** | **Analyte** | **YI** | **CI lower** | **CI upper** |
| --- | --- | --- | --- | --- | --- | --- |
| Ng et al 2009 | Singapore | Plasma | EGF | 3.70 | 2.25 | 5.14 |
| Chow et al 2010 | Singapore | Plasma | EGF | -0.52 | -1.31 | 0.27 |
| Current study | Sri Lanka | Serum | EGF | -0.92 | -1.66 | -0.17 |
| EGF (p value = 5.54E-01) | |  |  | 0.65 | -1.51 | 2.81 |
| I^2^ = 93.80; heterogeneity p value = 1.00E-07 | | | | | | |
| Ng et al 2009 | Singapore | Plasma | Eotaxin | 1.62 | 0.61 | 2.63 |
| Chow et al 2010 | Singapore | Plasma | Eotaxin | -0.92 | -1.72 | -0.11 |
| Wauquier et al 2011 | Gabon | Plasma | Eotaxin | 0.38 | -0.17 | 0.92 |
| Current study | Sri Lanka | Serum | Eotaxin | -3.51 | -4.43 | -2.60 |
| Eotaxin (p value = 5.32E-01) | |  |  | -0.61 | -2.51 | 1.30 |
| I^2^ = 95.67; heterogeneity p value = 6.00E-15 | | | | | | |
| Ng et al 2009 | Singapore | Plasma | GM-CSF | -0.65 | -1.55 | 0.25 |
| Chow et al 2010 | Singapore | Plasma | GM-CSF | 0.11 | -0.67 | 0.89 |
| Wauquier et al 2011 | Gabon | Plasma | GM-CSF | -1.12 | -1.71 | -0.54 |
| Chirathaworn et al 2013 | Thailand | Serum | GM-CSF | -1.28 | -1.91 | -0.65 |
| Current study | Sri Lanka | Serum | GM-CSF | 0.18 | -0.55 | 0.91 |
| GM-CSF (p value = 6.36E-02) | |  |  | -0.58 | -1.20 | 0.03 |
| I^2^ = 73.11; heterogeneity p value = 4.96E-03 | | | | | | |
| Ng et al 2009 | Singapore | Plasma | HGF | 1.51 | 0.51 | 2.50 |
| Chow et al 2010 | Singapore | Plasma | HGF | -0.74 | -1.53 | 0.06 |
| Wauquier et al 2011 | Gabon | Plasma | HGF | 0.97 | 0.40 | 1.54 |
| Current study | Sri Lanka | Serum | HGF | -1.18 | -1.94 | -0.43 |
| HGF (p value = 8.40E-01) | |  |  | 0.13 | -1.11 | 1.36 |
| I^2^ = 90.56; heterogeneity p value = 5.82E-07 | | | | | | |
| Ng et al 2009 | Singapore | Plasma | IL-13 | -0.41 | -1.30 | 0.47 |
| Chow et al 2010 | Singapore | Plasma | IL-13 | 1.11 | 0.29 | 1.93 |
| Wauquier et al 2011 | Gabon | Plasma | IL-13 | -1.06 | -1.64 | -0.48 |

**Supplementary Table 3.** (continued)

| Chirathaworn et al 2013 | Thailand | Serum | IL-13 | -1.00 | -1.61 | -0.39 |
| --- | --- | --- | --- | --- | --- | --- |
| Current study | Sri Lanka | Serum | IL-13 | 0.43 | -0.30 | 1.16 |
| IL-13 (p value = 6.13E-01) | |  |  | -0.21 | -1.03 | 0.61 |
| I^2^ = 85.00; heterogeneity p value = 2.32E-05 | | | | | | |
| Ng et al 2009 | Singapore | Plasma | IL-1β | -0.41 | -1.29 | 0.48 |
| Chow et al 2010 | Singapore | Plasma | IL-1β | 0.22 | -0.56 | 1.00 |
| Wauquier et al 2011 | Gabon | Plasma | IL-1β | -1.13 | -1.72 | -0.55 |
| Chirathaworn et al 2013 | Thailand | Serum | IL-1β | -0.22 | -0.80 | 0.35 |
| Reddy et al 2014 | India | Plasma | IL-1β | -0.26 | -0.74 | 0.23 |
| Current study | Sri Lanka | Serum | IL-1β | 0.63 | -0.10 | 1.37 |
| IL-1β (p value =3.65E-01) | |  |  | -0.22 | -0.69 | 0.25 |
| I^2^ = 68.23; heterogeneity p value = 7.63E-03 | | | | | | |
| Ng et al 2009 | Singapore | Plasma | IL-8 | 2.17 | 1.07 | 3.28 |
| Chow et al 2010 | Singapore | Plasma | IL-8 | -1.34 | -2.17 | -0.50 |
| Wauquier et al 2011 | Gabon | Plasma | IL-8 | -1.40 | -2.00 | -0.79 |
| Lohachanakul et al 2012 | Thailand | Plasma | IL-8 | 0.67 | 0.03 | 1.32 |
| Chirathaworn et al 2013 | Thailand | Serum | IL-8 | -1.07 | -1.68 | -0.45 |
| Reddy et al 2014 | India | Plasma | IL-8 | -0.63 | -1.13 | -0.13 |
| Current study | Sri Lanka | Serum | IL-8 | -0.84 | -1.58 | -0.10 |
| IL-8 (p value = 2.93E-01) | |  |  | -0.40 | -1.16 | 0.35 |
| I^2^ = 88.31; heterogeneity p value = 2.54E-09 | | | | | | |
| Ng et al 2009 | Singapore | Plasma | IL-5 | -0.81 | -1.72 | 0.10 |
| Chow et al 2010 | Singapore | Plasma | IL-5 | -0.23 | -1.01 | 0.55 |
| Wauquier et al 2011 | Gabon | Plasma | IL-5 | -0.24 | -0.78 | 0.31 |
| Chirathaworn et al 2013 | Thailand | Serum | IL-5 | -0.92 | -1.53 | -0.32 |
| Reddy et al 2014 | India | Plasma | IL-5 | -0.26 | -0.75 | 0.23 |
| Current study | Sri Lanka | Serum | IL-5 | 1.76 | 0.98 | 2.54 |
| IL-5 (p value = 7.12E-01) | |  |  | -0.13 | -0.80 | 0.55 |
| I^2^ = 84.15; heterogeneity p value = 7.30E-06 | | | | | | |
| Ng et al 2009 | Singapore | Plasma | RANTES | 0.42 | -0.47 | 1.30 |
| Chow et al 2010 | Singapore | Plasma | RANTES | -1.29 | -2.12 | -0.46 |
| Current study | Sri Lanka | Serum | RANTES | -2.29 | -3.11 | -1.48 |
| RANTES (p value = 1.72E-01) | |  |  | -1.06 | -2.59 | 0.46 |
| I^2^ = 89.84; heterogeneity p value = 5.30E-05 | | | | | | |
| Ng et al 2009 | Singapore | Plasma | TNF-α | 0.04 | -0.83 | 0.92 |
| Chow et al 2010 | Singapore | Plasma | TNF-α | -0.77 | -1.57 | 0.03 |
| Wauquier et al 2011 | Gabon | Plasma | TNF-α | -0.18 | -0.72 | 0.37 |
| Lohachanakul et al 2012 | Thailand | Plasma | TNF-α | 0.43 | -0.21 | 1.07 |
| Chirathaworn et al 2013 | Thailand | Serum | TNF-α | -1.27 | -1.90 | -0.65 |
| Reddy et al 2014 | India | Plasma | TNF-α | -0.28 | -0.77 | 0.20 |

**Supplementary Table 3.** (continued)

| Current study | Sri Lanka | Serum | TNF-α | -1.84 | -2.62 | -1.05 |
| --- | --- | --- | --- | --- | --- | --- |
| TNF-α (p value = 5.36E-02) | |  |  | -0.54 | -1.09 | 0.01 |
| I^2^ = 79.44; heterogeneity p value = 5.63E-05 | | | | | | |
| Ng et al 2009 | Singapore | Plasma | VEGF | -2.40 | -3.55 | -1.25 |
| Chow et al 2010 | Singapore | Plasma | VEGF | 3.12 | 2.07 | 4.17 |
| Wauquier et al 2011 | Gabon | Plasma | VEGF | -1.78 | -2.42 | -1.14 |
| Current study | Sri Lanka | Serum | VEGF | -0.50 | -1.24 | 0.23 |
| VEGF (p value = 7.05E-01) | |  |  | -0.40 | -2.47 | 1.67 |
| I^2^ = 95.75; heterogeneity p value = 3.18E-15 | | | | | | |

**Supplementary legends**

**Supplementary Figure 1.** Schematic diagram on the classification of clinical specimens. One hundred and seven clinical specimens were included in this study. Specimens were validated by RT-PCR, virion-based and peptide-based ELISA assays and sub-categorized into 2 groups CHIKV positive; Non-CHIKV. The CHIKV group was further sub-categorized according to the RT-PCR results into CHIKV PCR positive group (n = 71) and CHIKV PCR negative group (n = 28). Samples were considered CHIKV RNA positive if crossing point values were higher than cycle 35.Using a PCR value of cycle 25 as threshold, 46 CHIKV PCR positive patients were classified into high viral load (HVL) group (PCR value < cycle 25) and 25 patients were classified into low viral load (LVL) group (PCR value > cycle 25). Samples from the 8 patients that are triple-negative for the RT-PCR, virion-based and peptide-based ELISA analyzes were used as controls in this study.

**Supplementary Figure 2.** Cytokine and chemokine profiles in CHIKV PCR positive patients. CHIKV PCR positive patients (n = 71) were further classified into LVL (n = 25) and HVL (n = 46). Levels of cytokines and chemokines data were presented by 2-way hierarchical clustering. Each colored well in the heat map represents the relative levels of each cytokine. Green indicates low expression and red indicates high expression.

**Supplementary Figure 3.** Forest plots of immune mediators that were not significantly elevated in different CHIKF patient cohorts. *A*, pro-inflammatory cytokines and *B*, chemokines and *C,* growth factors. Dotted line represents no differences in the mean of the healthy controls and CHIKF patients. Diamond represents the combined effect size for each mediator.


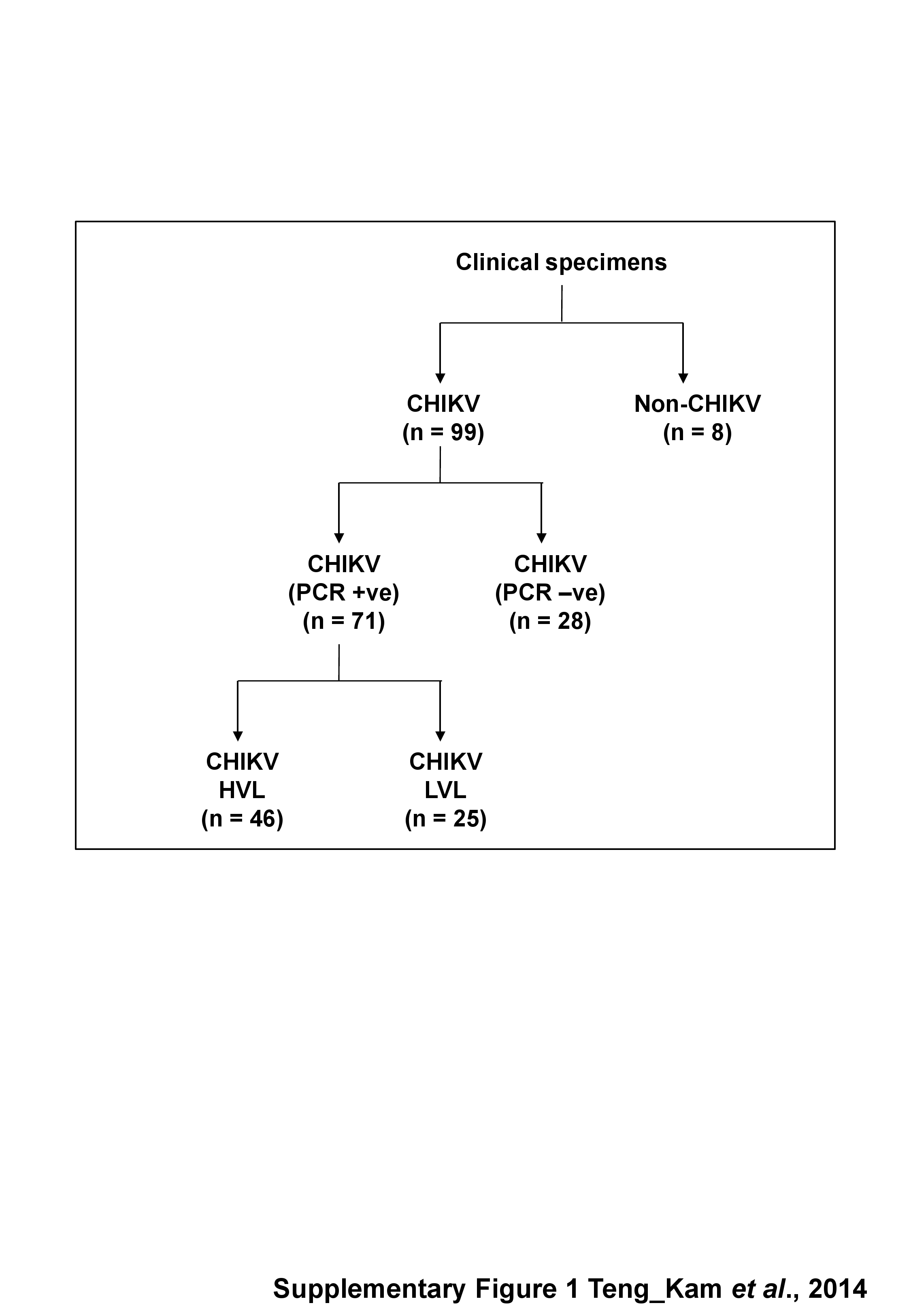


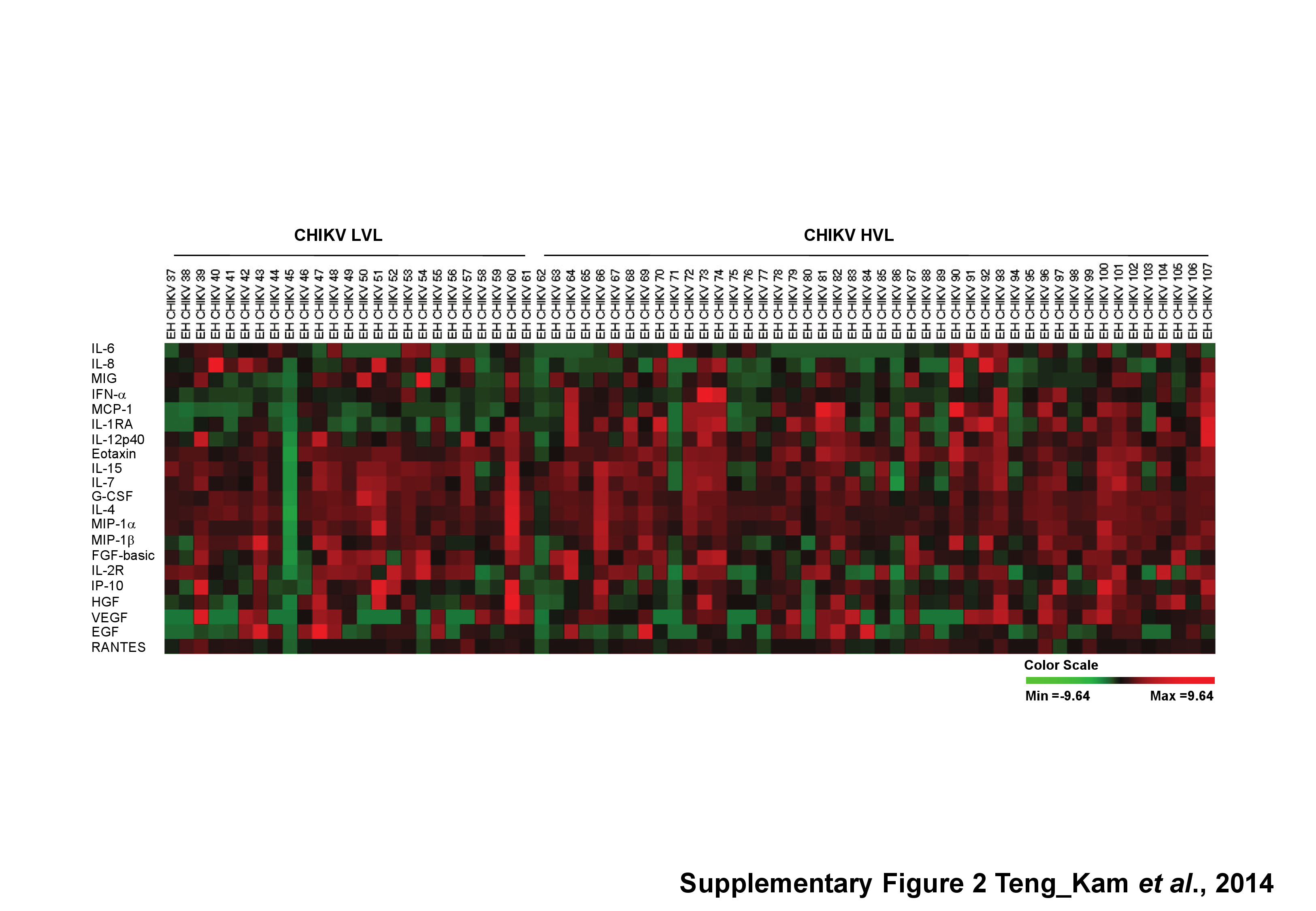


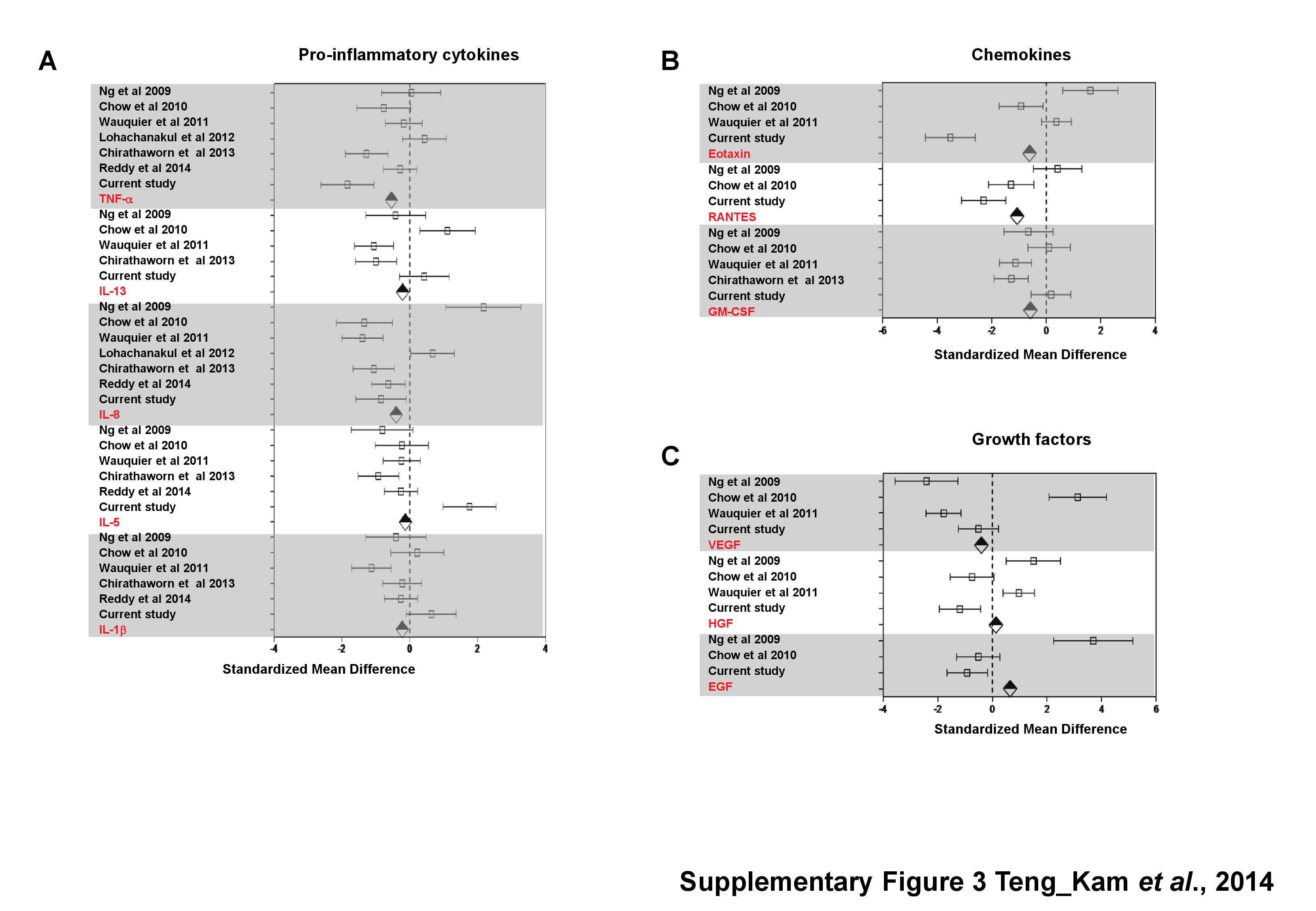

Supplement: Supplementary Data [file supp_jiv049_jiv049supp.doc]
