# Supplementary material for: A Systematic Meta-analysis of Immune Signatures in Patients With Acute Chikungunya Virus Infection
Source: J Infect Dis. 2015 Jan 29;211(12):1925–35. doi: 10.1093/infdis/jiv049 (PMC4442625; doi:10.1093/infdis/jiv049)
Supplement: Supplementary Data [file supp_jiv049_jiv049supp_tables.doc]

**Supplementary Table 4.** Expression profiles of circulatory immune mediators in DV-infected patients from 22 independent published studies. The cytokines, chemokines and growth factors that were significantly elevated during the acute phase of the infection are presented in the table.

| Origin |  | Immune mediators | |  | Reference | |
| --- | --- | --- | --- | --- | --- | --- |
| Venezuela |  | TNF-α  IL-6 | IL-12  IL-17 |  | (2014) Virology, 452-453, 42–51 | |
|  |  | IL-10 |  |  | (2012) AJTMH, 86(2), 341–348 | |
|  |  | TNF-α |  |  | (2005) Cytokine, 30, 359-365 | |
|  |  |  |  |  |  | |
| Colombia |  | IFN-γ  IL-6 | TNF-α |  | (2008) AJTMH, 79(5), 673–677 | |
|  |  | IFN-γ | IL-6 |  | (2008) R da SB de MT 41(1), 6-10 | |
|  |  |  |  |  |  | |
| Costa Rica |  | TNF-α  IL-8 | IL-6 |  | (2004) Cytokine, 27, 173-179 | |
|  |  |  |  |  |  | |
| Cuba |  | IL-10 |  |  | (2004) JMV, 73, 230–234 | |
|  |  |  |  |  |  | |
| Brazil |  | IP-10  IL-1Ra | MIP-1β |  | (2012) Mem Inst Oswaldo,107, 48-56 | |
|  |  | IFN-γ  TNF-α  IL-6  MIP-1β  IL-10  MCP-1 | IL-7  IL-1β  IL-13  IL-2  IL-4 |  | (2008) BMC Infect Dis, 8, 86 | |
|  |  |  |  |  |  | |
| India |  | IFN-γ | TNF-α |  | (2014) Int J of Infect Dis, 18, 68–72 | |
|  |  | IL-17 |  |  | (2013) J Clin Immunol, 33, 613–618 | |
|  |  | IFN-γ  IL-6 | TNF-α  IL-8 |  | (2010) PLOS ONE, 5(1), e8709 | |
|  |  |  |  |  |  | |
| Indonesia |  | TNF-α  IL-1β | IL-1Ra |  | (2003) Eur. Cyto Netw,14, 172–177 | |
| **Supplementary Table 4. (continued)** | | | | | |  |
| Origin |  | Immune mediators | |  | Reference | |
| Sri Lanka |  | IL-10 |  |  | (2013) BMC Infect Dis, 13, 341 | |
|  |  | TNF-α  IL-6  TGF-ß  MIP-1ß | IL-17  IP-10  IL-10 |  | (2012) PLoS ONE, 7(11), e50387 | |
|  |  |  |  |  |  | |
| Vietnam |  | IFN-γ  IL-6 | TNF-α  IL-10 |  | (2004) JID, 189, 221–32 | |
|  |  |  |  |  |  | |
| Singapore |  | IFN-γ  MIP-1β | IL-1β Eotaxin |  | (2012) PNTD, 6(11), e1887 | |
|  |  | IL-6  IL-8  IL-10  IP-10  IL-13 | IL-12  FGF PDGF  IL-4 |  |  | |
|  |  | MCP-1  IP-10 | MIP-1α MCP-2 |  | (2011) BMC Infect Dis, 11, 209 | |
|  |  |  |  |  |  | |
| Malaysia |  | IP-10  MIP-1β | MCP-1 G-CSF |  | (2012) PLoS ONE, 7(12), e52215 | |
|  |  |  |  |  |  | |
| Taiwan |  | IFN-γ  IFN-α | IL-10 |  | (2007) TRSTMH, 101, 1106—1113 | |
|  |  | IFN-γ  TNF-α  IL-6 | IL-10  MIF |  | (2006) AJTMH, 74(1), 142–147 | |
|  |  |  |  |  |  | |
| Gabon |  | IFN-γ  IL-6 | SDF-1α MIF |  | (2010) BMC Infect Dis, 10, 356 | |
|  |  | IL-17  IFN-α2  IL-1Ra  IL-2ra  IL-13  MCP-1  IP-10 | RANTES IL-16  IL-7  IL-12p40, G-CSF, GM-CSF, VEGF-A |  |  | |
|  | | | | | |  |
